# Crimmigrating Narratives: Examining Third-Party Observations of US Detained Immigration Court

**DOI:** 10.1017/lsi.2022.16

**Published:** 2022-06-30

**Authors:** Christopher Levesque, Jack DeWaard, Linus Chan, Michele Garnett McKenzie, Kazumi Tsuchiya, Olivia Toles, Amy Lange, Kim Horner, Eric Ryu, Elizabeth Heger Boyle

**Affiliations:** University of Minnesota; James H. Binger Center for New Americans at the University of Minnesota Law School; Minnesota Population Center; Department of Sociology and Graduate Faculty, Minnesota Population Center, University of Minnesota; Hubert H. Humphrey School of Public Affairs, Institute on the Environment and the Life Course Center; University of Minnesota; Detainee Rights Clinic, University of Minnesota; Advocates for Human Rights; Dalla Lana School of Public Health in the Social and Behavioural Sciences Division, University of Toronto; University of Minnesota - Twin Cities; Advocates for Human Rights; University of Minnesota - Twin Cities; University of Minnesota - Twin Cities; Minnesota Population Center, University of Minnesota

## Abstract

Examining what we call “crimmigrating narratives,” we show that US immigration court criminalizes non-citizens, cements forms of social control, and dispenses punishment in a non-punitive legal setting. Building on theories of crimmigration and a sociology of narrative, we code, categorize, and describe third-party observations of detained immigration court hearings conducted in Fort Snelling, Minnesota, from July 2018 to June 2019. We identify and investigate structural factors of three key crimmigrating narratives in the courtroom: one based on threats (stories of the non-citizen’s criminal history and perceived danger to society), a second involving deservingness (stories of the non-citizen’s social ties, hardship, and belonging in the United States), and a third pertaining to their status as “impossible subjects” (stories rendering non-citizens “illegal,” categorically excludable, and contradictory to the law). Findings demonstrate that the courts’ prioritization of these three narratives disconnects detainees from their own socially organized experience and prevents them from fully engaging in the immigration court process. In closing, we discuss the potential implications of crimmigrating narratives for the US immigration legal system and non-citizen status.

## INTRODUCTION

In the Fort Snelling, Minnesota, detained immigration court, it is common to see a detainee alone, wearing an orange jumpsuit with ankles shackled underneath the table. More than half of detainees sit alone without an attorney by their side, a common trend in immigration courts across the country (Transactional Records Access Clearinghouse [TRAC] 2017b). Unlike in criminal courts, immigration proceedings in federal courts across the United States and its territories do not provide the right to a public defender at the court’s expense (Torrey 2015). This is especially troubling when people are detained, and the burden falls on them, not on the state, to prove they should be released on bond and granted relief (Cade 2015; Chan 2021). These civil proceedings afford none of the basic protections that accompany criminal legal proceedings, depriving migrants of their life, liberty, and/or property (Golash-Boza 2015). This deprivation constitutes two forms of legal banishment: detention and deportation or what immigration procedure calls “removal.”

This article builds on a “sociology of narrative” theorized by Patricia Ewick and Susan Silbey (1995). We put their work into conversation with crimmigration, an area of overlap between immigration and criminal law that defines non-citizens’ experience of the country’s legal system (Stumpf 2006; see also Cházaro 2016; Vázquez 2017; García Hernández 2018, 2019). Using this framework, we ask and answer the following questions: which narratives shape and define the immigration court process and what happens when the immigration court system privileges certain narratives over others?

This research adds to the growing number of empirical studies of US immigration court in the past decade (Eagly 2015; Eagly and Shafer 2015, 2020a, 2020b; Ryo 2016, 2018, 2019a; Ryo and Peacock 2018; Asad 2019). Our questions also resonate with theoretical research covering both the criminalization of immigration enforcement and the “immigrationization” of penal systems in the global North (Brandariz 2021). To answer our questions, we use third-party immigration court observations to construct and apply a theoretical concept of what we term “crimmigrating narratives,” defined as selective tellings that categorize individuals as criminals based on their non-citizen status. Given Linus Chan and Kathryn Burkart’s (2019) argument that non-legal actors help shape, critique, and determine the court’s legitimacy and the morality of the immigration law enforcement system itself, analyzing third-party perspectives can help readers and practitioners better understand how the public perceives immigration court in action.

We find that respondents’ lack of full inclusion in the immigration court process is less of a shortcoming than a feature of immigration law as it currently exists. We identify three crimmigrating narratives—based in threat, deservingness, and impossibility—and analyze how courtroom interactions reflect, establish, and carry out power relations among legal actors. We also show how the court process reveals the relationship between these narratives and codified law. This informs a broader discussion about the law and how it creates subjects that do and do not belong within the nation-state’s boundaries (Ngai 2004).

## BACKGROUND AND THEORETICAL FRAMEWORK

### A Court in Collapse

To the American Bar Association (ABA) (2019, 15), the US immigration court system is “irredeemably dysfunctional and on the brink of collapse.” At root of the court’s insolvency is its inability to promise fair and unbiased treatment for non-citizens, a lack of judicial independence that fails to safeguard against political interference in immigration law, an under-resourced and overworked staff, and a backlog of immigration-related cases surpassing 1.6 million in 2021 (TRAC 2022). This backlog has more than quadrupled in the past decade, signaling the court system’s inability to keep pace with heightened immigration enforcement and maintain legitimacy in the realm of American jurisprudence (Esthimer 2019).

The ABA’s report highlights two systemic issues that have affected immigration law’s overall legitimacy for many years. First, judicial decision making and resulting case outcomes continue to depend on the assigned immigration judge (IJ) (Ramji-Nogales, Schoenholtz, and Schrag 2007; TRAC 2017a). Research demonstrates that judicial independence, training, and supervision create openings for implicit bias to influence decisions (Marouf 2011). In addition, IJ decision making unfolds against a complex and potentially fraught backdrop of individual, situational, and contextual factors (Eagly and Shafer 2015; Miller, Camp Keith, and Holmes 2015; Asad 2019; Ryo 2019a, 2019b). The second systemic issue is “public skepticism and a lack of respect for the immigration court process” itself (ABA 2019, 15). A Pew Research Center (2019) report found that a majority of self-identified Democrats and Republicans say it is important to increase the number of judges overseeing asylum cases, highlighting the overall public skepticism over the US immigration court system and its capacity to mete out justice. Socio-legal scholarship also suggests that increased cynicism toward immigration law revolves around beliefs “that the legal system is punitive despite its purported administrative function, that legal rules are inscrutable by design, and that legal outcomes are arbitrary” (Ryo 2019b, 108; also see Ryo 2017).

A key reason for cynicism is that the immigration court process is far from orderly. IJs are overworked (Lustig, Delucchi, and Tennakoon 2008), job turnover has risen (TRAC 2020), and the hiring pace for new IJs remains insufficient to keep up with the court system’s burgeoning workload (TRAC 2019a). Past efforts to improve IJ quality and performance have fallen short, with immigration lawyers questioning whether the court’s overseeing agency—the Executive Office for Immigration Review (EOIR)—sufficiently addresses these concerns (American Immigration Lawyers Association 2013; Transactional Records Access Clearinghouse 2008). Technology and infrastructure often break down and impede the respondent’s already-limited due process protections, delaying a trial’s end well beyond the established date (Eagly 2015; ABA 2019).^1^ Meanwhile, in the past thirty years, immigration enforcement, detention, and deportation have effectively sped up, leading to new forms of criminalization and banishment that are straining the court’s resources (Golash-Boza 2012).

Another criticism is that detainees face major challenges in securing defense counsel. These include financial limitations, underfunded pro bono defense services, and private attorneys who are disincentivized from providing representation to those with time-consuming and difficult cases (Markowitz 2009). Such factors have contributed to a representation rate of roughly 30 percent between 2015 and 2017, which is much lower than the rate for those in non-detained proceedings (TRAC 2017b). The lack of representation is a concerning trend, given that access to legal services and representation is an important tenet of accessing justice (Sandefur 2015). For instance, retaining an immigration attorney increases the likelihood of judges granting bond (Ryo 2018). IJs also tend to view those with attorney representation as being less dangerous than those who are *pro se* (self-represented), although Emily Ryo (2019a) finds that IJs are more likely to consider Central American detainees a threat than others, regardless of their having counsel. In the language of our analysis, legal representation has the potential to play an important role in challenging crimmigrating narratives that exist in immigration court, with the increased potential to understand which stories are heard and why certain narratives are effective.

### Crimmigration, Narratives, and Crimmigrating Narratives

Past legislation targeting non-citizens—as well as the overall genealogy of immigration restriction—has created what Juliet Stumpf (2006) refers to as “crimmigration,” a form of law that merges the punitive powers of immigration and criminal law. US legislation such as the 1988 Anti-Drug and Abuse Act and the 1996 Illegal Immigration Reform and Immigrant Responsibility Act (IIRAIRA) have expanded the range of deportable offenses to include more felony and misdemeanor crimes.^2^ Recent “zero-tolerance” policies also have allowed immigration enforcement to priori-tize the detention and prosecution of non-citizens whose only crime involves unauthorized entry (Hagan, Castro, and Rodriguez 2010; Department of Justice 2018).

A growing portion of crimmigration studies has focused on the structural vulnerabilities of immigration detention (Tsuchiya et al. 2021). In detention centers, many non-citizens remain behind bars for long periods, often for reasons tied to local/private prison contract incentives. Denise Gilman and Luis Romero (2018) find that when there are fewer immigrants in a detention center, Immigration and Customs Enforcement (ICE) tends to set higher bond amounts for each individual. Similarly, the immigration system falls short in considering non-citizens’ financial ability to pay bond amounts, calling into question the constitutionality of immigration detention (Tan and Kaufman 2017). Particularly for detained respondents, crimmigration “legitimizes greater expenditures of state power to control the liberty of the non-citizen using criminal and immigration enforcement tools” (Stumpf 2013, 60).

Central to our analysis of crimmigration is Ewick and Sibley’s (1995) theoretical framework of the “sociology of narrative.” In their conceptualization, a narrative differs from a chronicle, such as a list of events that may have taken place in a respondent’s life. Instead, a narrative is characterized by a selective telling of ordered events that are related to both one another and an overarching idea or structure. In other words, there is an explicit or implied meaning to the telling of events. Although they identify three features of narrative in social science research (that is, as an object, product, or method), our analysis focuses solely on narratives as an object of study. Specifically, we explore the development and effect of what we call “crimmigrating narratives,” defined as selective tellings that categorize individuals as criminals based on their non-citizen status. This framework relies on a particular definition of narrative as well as on an understanding of the relationship between narrative, society, and power.

The emergence of narratives in the US immigration system is not random. They arise from the law itself and are intimately tied to their social, historical, and institutional setting. In legal settings, Ewick and Sibley argue that narratives support power imbalances that contribute to ongoing structural inequalities. To gain an understanding of these narratives, analysis must consider when certain types of narratives are demanded, what kind of narratives are deemed useful and appropriate, how narratives are expected to be shared, and why certain narratives are strategic or effective. By getting at these questions, we gain an understanding of the role that narratives play in immigration court specifically.

Prior research explores how legal actors employ narrative strategies to shape and mold perceptions of an individual’s character. These strategies are important for transforming the legal process or justifying the law’s outcomes. Focusing on the asylum process, legal actors construct immigrants’ narratives of persecution in their asylum applications to appear logical, cohesive, and chronological in order to demonstrate their well-founded and rational fear of returning to their country of origin (Coutin 2003). For law enforcement, US Border Patrol agents use their own narratives to not only criminalize immigrants crossing the border but also to reaffirm the Border Patrol’s moral prerogative to coerce and control as a means of compassion and care (Vega 2018).

Our study similarly examines how legal actors employ narrative strategies but within US immigration court. Because the court process largely ignores the respondent’s relationship to a much broader system of non-citizen exclusion, the respondent must tell a particularized personal story to avoid deportation rather than a generalized one that speaks to broader structural concerns. This finding is reminiscent of John Conley and William O’Barr’s (1990) assertion that courtroom narratives are stationed between relational orientation and rule orientation. On the one hand, legal parties use strategy to determine how much to reveal or omit in regard to the litigant’s social status and position (relational orientation). On the other hand, they must decide how much to defer to legal rules and principles (rule orientation). In the context of a trial, the chosen narrative strategy can either subvert or legitimize the status quo (Ewick and Silbey 1995; see also Williams 1991; Hunter 2008; Englebrecht and Chavez 2014).

To illustrate when and how narratives emerge in immigration court, consider the various stages of the hearings process. Starting with a master calendar hearing (or hearings), the IJ confers with both sides to determine a schedule for submitting evidence as well as a date for the case’s final merits hearing and testimony. During the master calendar hearing, which usually lasts fifteen minutes or less, the IJ asks the respondent to plead to each charge, correct any errors based on factual allegations in the charging document (also called the “Notice to Appear”), and decide which types of immigration relief to request, such as asylum or cancellation of removal. This process is quick and moves without much context, as the IJ does not consider any substantive arguments during these types of hearings. However, the pleadings process still puts the respondent at a disadvantage as their criminal history and potential “danger” become facets of the underlying courtroom story.

If eligible, a detained respondent may request to be released on bond. In this hearing (separate from the master calendar hearings), the IJ must determine whether the respondent is a “danger to society,” a “flight risk,” or a “threat to national security” (Department of Justice 2021a). To do so, the IJ asks the respondent or their attorney questions about the former’s employment, criminal history, and ties to family in the United States. This stage often occurs to the respondent’s detriment, raising concerns about individual liberty and due process (Chan 2021). Because the law puts the burden of proof on the respondent to prove bond eligibility, they can remain in custody for prolonged periods, sometimes six months or longer for so-called “criminal aliens” (Anello 2014).^3^

Testimony and cross-examination occur later during the merits hearing, when the IJ also decides whether the respondent will be removed (deported) from the United States. This final stage can be the first time a respondent fully explains their story and identifies the personal equities of their case—that is, any characteristics of the respondent that an adjudicator may view as positive—which includes having old convictions, long duration of residence, and ties to family in the United States. Doing so helps ensure fair and proportional application of the law toward individual respondents, even when there are civil or minor criminal violations on the respondent’s record. However, today’s US immigration system does not adequately consider equities, even at the merits stage (Cade 2015), which is due to amendments to the US immigration code limiting the discretion of IJs in considering social and personal factors in their decision (Salyer 2020). It is also because the Department of Homeland Security (DHS) enforcement priorities push ICE prosecutors to focus on criminal records rather than on equities (Koh 2017).

Within these concerns about judicial and prosecutorial discretion resides the issue of the law itself. Crimmigration law broadened what counts as “criminal history” in an immigration context, as many removals since the IIRAIRA have been linked to immigration crimes, traffic offenses, or other misdemeanors (Jain 2015; DHS 2019). Today, it renders a non-citizen’s criminal history “as an indiscriminate marker of undesirability” (Cade 2015, 661), while courts delay or even ignore substantive consideration of a case’s other mitigating factors. Discussions of the respondent’s criminal history can take place during any hearing, including master calendar and bond hearings. Thus, crimmigrating narratives often enter the legal space well before respondents’ own narratives do. As they take hold, it becomes difficult for respondents to question why they are in removal proceedings to begin with. The only options to end proceedings before the merits hearing are to use legal technical arguments that do not explicitly address equities or request removal. Analyzing crimmigrating narratives in these early stages can help explain how a respondent’s story begins to map onto statutory interpretation.

We argue that the court process prioritizes crimmigrating narratives that individuate the non-citizen and, like other hegemonic narratives, “reproduce, without exposing, the connections of the specific story and person to the structure of relations and institutions that make the story plausible” (Ewick and Silbey 1995, 214). These narratives are founded in rule-based orientations of the law and aim to criminalize individuals based on their immigration status.^4^ Here, we place crimmigration in conversation with previous scholarship on immigrant narratives—namely, depictions of immigrants as “threats” (Chavez 2008), “deserving” (Willen 2012; Shiff 2021), and/or “impossible subjects” (Ngai 2004)—to show how they limit respondents’ ability to transverse their perceived and assigned social roles in court.

### Conceptualizing Threat, Deservingness, and Impossibility

In his discussion on threat narrative, Leo Chavez (2008) analyzes how media and political discourse constructs and maintains myths about Latinos in the United States, drawing attention to nativist fears of crime, demographic change, and the erosion of “mainstream” American culture as a result of Latino migration to the United States. Using this definition, threat narratives portray certain immigrants as criminals morally incompatible with the receiving state, its people, and its laws. These types of narratives had long existed in Congress, whose statutory provisions have historically defined certain people as *per se* or probable threats. In court, the IJ and prosecutor’s scrutiny of criminal history result from and reinforce those statutory narratives. Governments typically label certain social groups as threats: since the September 11 attacks, immigration enforcement, detention, and deportation have sped up six-fold, with the dual intent to categorize Muslim- and Arab-Americans as “terrorists” and Latinos as “criminals” (Golash-Boza 2012). As Justin Pickett (2016, 103) argues, the ethnicity-coded issue of immigrant “threats” allows for “the veiled expression of broader anti-Latino sentiments” as well as increased support for immigration enforcement.

In court, threat narratives typically appear during immigration bond hearings. Empirical research confirms that IJs are less likely to grant bond to detainees with a criminal history (Ryo 2016), particularly Central Americans with felony and violent crime convictions (Ryo 2019a). Despite little proof that crime is linked to immigration, threat narratives demonstrate how detainees’ criminal histories compound their immigration status (Jiang and Erez 2018). Some non-citizens may have committed immigration violations—such as an unauthorized entry into the United States—that are either civil violations that do not result in criminal sanctions or low-level crimes that result in little to no criminal penalties. They may be removable due to crimes that US immigration considers to be “aggravated felonies” and/or “crimes of moral turpitude.” These crimes range from violent offenses (for example, murder, domestic abuse, aggravated assault) to smaller criminal offenses (for example, petty theft, public intoxication, drug possession). These expanded definitions of removability create openings for threat narratives to hold sway in immigration court proceedings (Stumpf 2006; García Hernández 2018). Criminalizing signs and indicia in the courtroom also reinforce threat narratives, where there are clearly visible security checkpoints, airlock entry systems, and armed guards, all of which “add up to a punitive atmosphere that does not differ from criminal prisons” (Solomon 2005, 9–10).

Another type of crimmigrating narrative concerns deservingness. Like threat narratives, deservingness relies on myth building and the criminalization of certain groups. However, it also focuses on who can be culturally, politically, and morally included in the United States in spite of their perceived transgressions of the law. To be portrayed as deserving, respondents’ personal stories must conform to a moral sense of belonging that mitigates their perceived “danger to society.” IJs ascertain a respondent’s deservingness in a number of ways, including the respondent’s strong family and community support, their fear of return, and their commitment to their own rehabilitation. Focusing on these characteristics, however, may also obscure more complicated narratives outside of the law that make up the respondent’s social reality. Similar to past research on immigrant rehabilitation services and penal power, deservingness narratives in immigration court require non-citizens to “participate in public dramatizations of their criminal stigma”—to revisit their own criminal histories—in exchange for the IJ granting bond or relief from deportation (Guzman 2020, 681).

Deservingness in the immigration context has its roots in the US assimilation movements of the early twentieth century (Ngai 2004), asylum (Shiff 2021), and debates over entitlements to social services including health care (Willen 2012). In these contexts, deservingness does not simply rest only on the non-citizen’s ability to tell their story. It also traces back to the law’s conception of who belongs and how adjudicators identify and prevent undue hardship to the respondent and their family. For instance, research on US asylum officers shows that they routinely understand deservingness as something based on non-citizens’ perceived traits, such as their gender, sexuality, or religious identity. These perceptions often bump up against codified definitions of asylum eligibility—moments of mismatch between personal belief and codified law that are called “encounters of ordinary discordance” (Shiff 2021, 339). These moments also happen in immigration court, as IJs grapple with their perceptions of who deserves to stay in the United States (Asad 2019). And while IJs may see an alignment between a respondent’s story and legal notions of deservingness, their own stereotypes and biases may cause skepticism about that story’s credibility (McKinnon 2009; Rempell 2010; Shiff 2021).

Drawing moral boundaries can exclude those who are not deserving, which is a central tenet of US immigration legal history (Salyer 1995; Law 2010; Kang 2017). Historians note that immigration law has always made “foreigners” (Parker 2015), “aliens” (Lew-Williams 2018), and “inmates” (Hernández 2017) by political design. This history underscores a third crimmigrating narrative of impossibility. Impossible narratives promote “illegality” as a marker of social difference, barring certain immigrants from US citizenship and foreclosing access to their legal rights. Impossible subjects, to use Mae Ngai’s (2004, xxiii) term, comprise a caste group “categorically excluded from the national community” due to their unauthorized legal status. The history of US immigration demonstrates that illegality itself is fluid and that the law makes and unmakes so-called “illegal aliens” based on social categories such as country of origin, gender, and race.^5^ Just as structural racism evolves with the law, the definition of impossibility is ever changing to maintain sovereign borders, produce new categories of racial difference, and naturalize social relations. While the line between “legal” and “illegal” remains blurred, immigrant exclusion based on impossibility continues in current codified law (Menjívar 2006; Menjívar and Abrego 2012).

Impossible narratives emerge in the courtroom when the government sees the respondent as statutorily ungovernable and, thus, an insoluble social problem (De Genova 2002). Adjudicators equate illegality with criminality, as the law defines an “illegal alien” in one of two ways: either they are unlawfully present in the United States or they have committed a deportable crime. In either case, the only way to resolve a respondent’s impossibility is through their legalization of status or expulsion. These options are narrow by design, which shows that impossibility is less of a shortcoming than a feature of immigration law itself. These narratives also arise when the court process—with its long delays, complex arguments, and insufficient interpreter services—assumes a non-citizen’s inclusion as both impractical and impossible (Barak 2021). As a result, this process systematically shuts them out from fully engaging during proceedings.

### Crimmigrating Narratives from a Third-party Perspective

The US immigration system has long been “shrouded in secrecy and bureaucratic barriers” and vulnerable to political interference (Ryo 2019b, 98; Office of the Inspector General 2021). Barriers create methodological challenges for immigration court researchers, and the EOIR’s data stewardship has recently been called into question (TRAC 2019b). Given the contradictory and clandestine nature of US immigration law, more creative, on-the-ground approaches to research are necessary. This need coincides with growing public distrust in the immigration court system (ABA 2019, 15), as the number of volunteer-led organizations observing and collecting data on immigration court has grown, especially since the first travel ban in 2017 (Wadhia 2019).^6^ Researchers have begun to analyze third-party immigration court observations, identifying multiple and overlapping areas of perceived structural vulnerability among detained non-citizens, such as financial insecurity, discrimination, and mental health (Tsuchiya et al. 2021). This growth in immigration court research follows a long tradition of sociologists and legal geographers observing US courts (Atkinson and Drew 1979; Walenta 2020) and international human rights practices (Weissbrodt 1982; Jeffrey and Jakala 2014). Advocacy-centered initiatives such as these highlight perceived dilatory and unjust practices in court and detention, contributing to an action-based project rooted in human rights fact-finding (Orentlicher 1990) and moral responsibility (Tait 2011). As immigration court observers have documented and witnessed using human rights models, this article develops a theoretical and empirical understanding of their observations.

By witnessing stories that respondents tell in court, third-party observers relate how the court process unfolds in real time using an open-ended and contextual framework. We focus on how legal actors scrutinize, discern, and apply moralizing judgments toward the respondent’s narrative and understanding of their social conditions. As a non-legal audience, observers take stock of moments where immigration court and the law imagine, and fail to imagine, the respondent’s perspective during trial. They also test their own expectations of justice and fairness against what they observe. In doing so, they construct “social spaces which, though they reveal themselves only in the form of highly abstract, objective relations, [make] the whole reality of the social world” (Bourdieu and Wacquant 1992, 231; see also Jiang and Erez 2018).

## DATA AND METHODS

This study uses open-ended form observations of the Fort Snelling, Minnesota, detained immigration court, with a primary focus on master calendar and bond hearings. The purpose of the study is to examine when, where, and how crimmigrating narratives arise based on the perceived threat, deservingness, and impossibility of non-citizen respondents. To analyze the observers’ responses and how they relate narrative in a courtroom setting, we relied on a coding scheme that incorporates themes deductively from previous crimmigration research and also allows for the emergence of these three narratives based on our coded results.

### The Human Rights Defender Project

The Advocates for Human Rights, the University of Minnesota Law School’s James H. Binger Center for New Americans, and Robins Kaplan LLP launched the Human Rights Defender Project (HRDP) following the mass mobilization of volunteers at US airports precipitated by the presidential travel bans.^7^ Designed to foster pro bono bond representation and bring the public into immigration court, the HRDP recruits, trains, deploys, and supports volunteers to monitor and document hearings; analyzes and reports on issues and trends surfaced through the monitoring and documentation; advocates with systems actors; and identifies and refers cases in need of representation to pro bono counsel (HRDP 2020). The HRDP engages trained volunteer observers to attend immigration court hearings and report on issues of concern, including, for example, the manner of arrest, access to counsel, the ability of individuals to raise defenses to deportation, family and community support, interpretation, as well as IJ and attorney engagement. To collect data, the observers used a two-page court observation form that captured both closed- and open-ended responses (see Appendix 2).^8^ For our analysis, we chose to code all open-ended questions, including concerns about procedure (for example, “were there any technical issues with interpretation?”); courtroom interactions (for example, “did the detainee’s attorney interact with the client?”); and criminal history (for example, “was a criminal history mentioned by either the court or lawyers?”). A full table of these questions is included in Appendix 1.

The HRDP typically schedules two volunteers per observation shift, each lasting 1.5 hours in either the morning or afternoon, two to four days a week. Per the EOIR guidelines, public observation may take place only during master calendar and bond hearings and during final merits hearings with the IJ and respondent’s consent.^9^ Because master calendar and bond hearings are easier to publicly access, the HRDP observations almost exclusively focus on those two types. Observers are not the judge, counsel, or respondent, nor are they relations or even acquaintances of these legal actors. They are aware that hearings move quickly with limited information and that, in almost every case, they will not witness the final written or oral decision of the judge. Therefore, observers are not required to provide responses to each open-ended question but, instead, select questions that capture the relevant details of each hearing. A large number of observers first heard about the project via their church, synagogue, or other faith-based organization based in the Twin Cities. Additional volunteers learned about the project via the University of Minnesota or local human rights organizations. The authors of this article began collaborating with the HRDP partners at the beginning of 2018 to aid in data cleaning and collection.

The HRDP’s unique data raise at least two important considerations of our work in this article. First, in the absence of random selection, third-party immigration court observers are likely highly selected (on education, political affiliation, support for immigration, geography, and proximity to immigration court, and so on). We recognize that observations have the potential for bias because of observers’ pro-immigration stance as well as their alignment with the HRDP’s advocacy goals. Second, and as a consequence, the resulting observational data and associated findings may not be generalizable to the broader US population or fully representative of other immigration courts in the United States. While these concerns are valid, they are also outweighed by the need for new knowledge and public awareness about the immigration court system and how it functions. Recalling Howard Becker’s (1998, 87) critique of random sampling, it should be noted that third-party immigration court observers can be viewed as unusual cases that are “likely to upset your thinking” by providing a unique opportunity to glimpse first-hand perceptions of procedural fairness in immigration court and coproduce knowledge for future research.

Analyzing data from the Fort Snelling court is important for a number of reasons. First, the Fort Snelling court’s jurisdiction is unique in that it handles cases from a number of states—North Dakota, South Dakota, and Minnesota—considered to be new and reemerging settlement gateways for recently arrived immigrants (Donato et al. 2008; Passel and Cohn 2016).^10^ Previous work shows that unauthorized immigrants are more susceptible to ICE apprehension in new gateway states and mostly rural parts of the Midwest, where labor market conditions have worsened, the Latino population has increased, and conservative politics have strengthened (Moinester 2018; Ryo and Peacock 2020). Second, the Fort Snelling court is important in that, compared to other courts, it hears a proportionally large number of removal cases involving respondents from outside Mexico and Central America. This is partly due to Minnesota’s status as a historically popular destination for refugees in the United States, particularly for the Hmong in the 1970s (Allen and Goetz 2010), Somalis in the 1990s (Boyle and Ali 2010; Abdi 2014), and Liberians today (Corrie and Randosevich 2013).

### Sampling Strategy

For this article, we focused our analysis at the hearing level. During July 2018 to June 2019, the HRDP conducted a total of 3,125 hearing observations among 168 volunteers. Given the large number of observations, we restricted our analysis to a random sample (n = 301) limited to 10 percent of the original dataset, stratified by IJ and question (see Appendix 1). These observations reveal details involving various legal actors—including the IJ, prosecutor, attorney, interpreter, and/or respondent—as well as the perspective of the third-party observer. Table 1 presents descriptive statistics indicating the distribution of hearing type, attorney representation, and interpreter usage. All hearings took place in person rather than over video. In total, ninety-six of the 168 volunteer observers were present in our sample. Of the three judge categories, there were fifty-three unique observers for Judge Option 1, fifty-two unique observers for Judge Option 2, and twenty-six unique observers for Judge Option 3. We stratified the sample in this way because court processes and outcomes often differ by IJ and because observers sometimes leave questions blank if they do not pertain to the hearing (Ramji-Nogales, Schoenholtz, and Schrag 2007). As such, we wished to capture a representative sample of court observations in the full data set. In order to keep track of each observation, we assigned each question response a corresponding case identification that concatenates the hearing date, form question, judge identifier, origin country code, and the last three digits of the respondent’s alien registration number.

### Data Analysis

We performed our data analysis using NVivo, data management software for qualitative data analysis. Text search queries were employed to retrieve key words and phrases to be included in the analysis. This process involved multiple rounds of searching for terms such as “crime,” “aggravated felony,” “moral turpitude,” and other words and phrases used in immigration court that also refer back to the prior literature on crimmigration (Stumpf 2006; Vázquez 2017; García Hernández 2018). Relying on our experiences as lawyers, human rights advocates, and social scientists, we identified key crimmigration themes based on 119 text queries, allowing for an interdisciplinary application that coded words or phrases from the ground up with larger core categories related to crimmigration in mind. For example, searching for more specific terms such as “battery” and “assault” often fell into the category of criminalization, contributing to a larger theme based on threat perceptions, which finally led to selecting codes in the core category of “threat narrative.” This follows the guidelines of axial coding—the process of constructing an inductive and deductive relationship between codes—to examine the relationships between categories and refine them by placing them within the context of three crimmigrating narratives (threats, deservingness, and impossibility) (Strauss and Corbin 1998). This approach bolsters our theoretical understanding of the relationship between court process and interactions from our initial codes and categories.

The co-authors met regularly to discuss any discrepancies and to establish consensus during the coding process. In doing so, we were able to integrate our own legal and lay perspectives on immigation courts to form the following categories: (1) familial and social ties; (2) legal status; (3) judicial decisions; (4) court processes and procedures; (5) language and interpretation; (6) legal representation; (7) perceptions and emotions; and (8) criminalization. By implementing an iterative coding design, this list of categories captured the tension between formal and informal lay understandings of the law, building upon previous studies that investigate lay jurisprudence and legal discourse (Conley and O’Barr 1990; Barton 2004).

## FINDINGS

As in most trial formats, immigration court requires strategic storytelling in order to prove a case. For that reason, it is often difficult for those without legal experience to follow what occurs during a hearing. Given that legal perplexities can obscure one’s understanding of a case, we first note the stark difference between those having attorney representation and those who are *pro se*—the “haves” and the “have nots” (Galanter 1974). While the “haves” often rely on counsel to develop a sound legal strategy (Eagly 2015; Ryo 2018), the “have nots” often lack the specific legal training to develop a narrative strategy that appears competent, coherent, and responsive to the judge. Lacking a strategist can develop into the further criminalization and subordination of the respondent during immigration proceedings, as the HRDP observation data make clear. For instance, during an observed master calendar hearing, an unrepresented Somali detainee explained that he previously “pleaded guilty to something he never should have” on the advice of his public defender, and he was now stuck in detention trying to find an immigration attorney to represent him. He asked the IJ for help appealing his prior conviction, and the IJ explained that they had no authority to do this.^11^

Sometimes, the narratives that respondents employ without counsel are given full or even additional consideration by IJs—in another master calendar hearing involving an unrepresented Mexican respondent, a HRDP observer notes that “the judge said [they] ‘like to give latitude to unrepresented people’ while doing bond hearings.” However, there are other instances when IJs are less open to a respondent’s story. In a bond hearing involving another Mexican respondent, the same IJ “asked questions irrelevant to immigration, prejudicial to [the] criminal case, listed statements not taken under oath, seemed to have a bias from the start,” and they also “asked questions that [an] attorney may/should have objected to.” The lack of legal protections for non-citizens in US immigration proceedings, such as the right to an attorney at the government’s expense, presents a roadblock for those navigating immigration proceedings on their own.

### Threat Narratives

Court observations noted how the legal process prioritizes threat narratives, especially during bond hearings. Similar to criminal court, IJs in these hearings consider not only whether the respondent poses a “danger to society” but also whether they are a “flight risk.” While they often rely on police reports as evidence of a non-citizen’s criminal history, those police rarely testify in immigration court. As such, IJs rely on these hearsay documents to make life-altering decisions, regardless of whether a respondent is charged or convicted of a crime (Holper 2014).^12^ Preliminary immigration hearings therefore involve an initial inventory of a non-citizen’s charges and convictions without the same criminal protections.

In one observation, the IJ denied bond to a respondent with driving-under-the-influence (DUI) and traffic-related charges because the IJ deemed those charges to be “very serious.” The IJ reached this conclusion despite the defense counsel’s argument that the DUI was a first-time offense and that the respondent “has decent grades in school and would finish out his education this fall.” These character assessments also take place during the master calendar phase: in one such hearing, the IJ made note of the respondent’s “lengthy criminal record,” saying that “a detailed description of the items on that record will need to be addressed in order to establish that the detainee is not a danger to the community.” These examples show the indelible mark criminality can have on non-citizens, even at the beginning of a case. They demonstrate what Daniel Kanstroom (2007, 5) calls “post-entry social control,” a mechanism that depicts non-citizens as criminal threats in a civil court. By extending the state’s carceral power, threat narratives privilege an eternal probation model that treats non-citizens as forever guests in the United States (Beckett and Murakawa 2012). The seriousness of one’s crimes remains paramount, and a respondent’s overall criminal history helps shape and determine an IJ’s decision. Therein lies a paradox: despite falling outside of the realm of criminal law, the state engages in a process that criminalizes non-citizens and often denies them of their life, liberty, and/or property (Golash-Boza 2012).

Politicized debates over immigration law and policy also inform how threat narratives operate. Like accounts of criminal history, these debates rely on stigmas of criminality to rationalize immigration enforcement and removal (García Hernández 2019, 113). In one hearing, an IJ denied bond to a Mexican respondent despite his parents and siblings having legal status in the United States. This decision was a result of the fact that he had two recent DUIs on his record. From the observer’s perspective, the ICE prosecutor was “dogged in pursuing bond denial and in countering detainee’s arguments.” He commented that plans for alcohol treatment “should have happened after the first DUI,” with the observer noting how “he seemed on the edge of sarcasm in his tone during his rebuttal of the detainee’s lawyer.”

At the time this hearing took place (November 2018), popular media—both via mass communication (for example, television, radio) and digital sources (for example, Twitter)—focused extensively on the so-called “wave of migrants” traveling in a “caravan” toward the United States’ southern border (Semple and Malkin 2018). In-between hearings, the observer commented on the prosecutor’s behavior as being unprofessional, hearing him say “something along the lines of ‘Bangladeshis’ entering the country which I had the impression he was saying were part of the ‘caravan’ of immigrants in the news currently.” The observation draws a link between the prosecutor’s in-court argument, which focuses on the respondent’s perceived threat, and myths regarding immigrant groups approaching and already within the United States (Chavez 2008; Davíla 2008; Massey and Pren 2012). It highlights how threat narratives attempt to link criminal stigma to a group’s perceived traits, all while blaming an individual’s crimes. Some observations note that IJs try to maintain neutrality and admonish prosecutor’s attempts to bring more overtly political characterizations of threat narrative into the fray of discussion, suggesting “a sense of balance in the discretion they have in following the law,” as one observation notes. However, the same observer writes that IJs still “generally tip the balance against the detainees because the system is tilted that way.”

### Deservingness Narratives

Contrary to threat narratives, deservingness narratives are grounded in a moral sense of belonging rather than in a so-called danger to society. They focus on whether or not a person should be allowed to stay in the country despite their perceived violations of the law. Assessing deservingness involves multiple criteria in the IJ’s decision. For bond hearings, familial and social ties as well as the length of stay in the United States, employment, and education are important factors in building a respondent’s personal story and potentially leading to their release from detention. Defense attorneys often file letters of support that describe their clients’ good moral character and confirm that their clients are not a danger to the community, regardless of their legal status. In one bond hearing, a Mexican respondent with a pending DUI charge submitted “more than thirty letters of support” corroborating the community ties he had made in the United States in the past twenty years. With the help of an attorney, the respondent described his alcoholism as a mental health issue that required professional help. His attorney reassured the IJ that a relative would connect the respondent to treatment and that many people pledged to give him rides during recovery. The IJ granted bond shortly thereafter.

This example demonstrates how a positive outpouring of support can be convincing evidence of a non-citizen’s deservingness to stay in the United States. With a narrative strategy in place, this respondent argued not only that he was not a danger to society but also that his belonging in American society depended on his commitment to recovery. Despite having strong community ties, respondents must still publicly revisit their criminal history in a non-criminal court setting (Beckett and Murakawa 2012; Guzman 2020). While this action can recriminalize the respondent, having counsel to strategize and present these narratives still benefits his or her chances of receiving bond and/or relief from deportation.

However, because the majority of respondents lack counsel, the storytelling often falls upon the “have nots.” In another bond hearing, an unrepresented Mexican respondent brought notes and reference letters as evidence of his good moral standing and ties to the community, specifically through his son. Here, the respondent’s story appeared to fit into a deservingness narrative, but it still required legal documents as corroboration: the IJ asked for a birth certificate as proof of relationship “to establish that his removal would be a hardship to his U.S. citizen —beyond the ordinary hardship of separation from parent.” The IJ also requested documentation proving that the detainee had been present with the child in the year prior to detention. Without an attorney to strategize for the case, the IJ remained skeptical of the respondent’s story.

When respondents lack evidence for strong social ties, judges and prosecutors may question the respondent’s ability to return to court after their custody release (Eagly and Shafer 2020a). For instance, during a bond hearing, a respondent’s attorney stated that their client “has fears of returning to his country,” while the DHS attorney argued that the respondent was a flight risk as “most of his family is in Mexico and he has lived in many [US] states : : : as if moving in order to find work makes one irresponsible and unlikely to follow up in court.” Here, the prosecution cited the respondent’s limited ties within the United States as proof that they will disappear when released from detention. This faults the individual and not the broader structural risks of returning to one’s so-called “home country.” The respondent had no criminal convictions, and yet, in this bond hearing, the prosecution assumed that the respondent would be unable to follow the law. After it was clear that a bond decision would not be made without more documentation surrounding the respondent’s arrest, their attorney asked for and was granted a continuance. What this observation highlights is that to be portrayed as deserving, respondents’ personal stories must conform to a certain moral framing but that framing still relates to the respondent’s perceived social ties and criminal history. This obfuscates more complex, extralegal narratives that constitute the respondent’s social world.

In other cases, the IJ may still choose to impart normative judgments after granting bond to the detainee. In one hearing, a prosecutor argued that a Mexican respondent was a danger to society “because of her drinking.” Even post-ruling, after the IJ decided they were not a danger and granted them bond, they still “talked to her about NOT drinking and driving,” bringing up the respondent’s moral responsibility as “a mother with children.” Even though positive bond outcomes can still lead to stern reprimands, this example shows that IJs do attempt to consider the complexity of a respondent’s story during bond consideration. In a separate case, a judge granted bond

despite driving-while-intoxicated convictions since no one was hurt and no one was driving erratically. He has directions to [either] not drive or seek treatment. Not a flight risk—long time in United States, citizen children.^13^

Additional observations detail how IJs make efforts to educate as well as cover basic procedural information during hearings without a defense attorney. In one bond hearing, the judge “seemed to look for options for the detainee” and “expressed concern regarding [the] detainee’s mistreatment while in the United States.” Once no apparent options were available, “[the judge] stated to the detainee, ‘I wish you the best. Good luck.’” Here, one can see how judges are unable to use the little discretion they have to see beyond the law’s interpretation of the respondent’s narratives. The law often still binds respondents, however deserving, to processes of removal and exclusion by virtue of their non-citizen status.

### Impossible Narratives

As it attempts to resolve the tension between deservingness and threat, immigration court can be both receptive and cold toward the respondents. Still, it often fails to separate the general from the particular of a respondent’s story. As respondents open up about their lives in front of the bench, immigration judges and lawyers hear them in a criminalizing space where, as one IJ put it, they are “doing death penalty cases in a traffic court setting” (Drummond 2019). Detachment and estrangement from respondents’ social worlds allow the state and its bureaucratic actors to render punishment in a non-punitive setting, and this is where impossible narratives reveal themselves. This issue is at the crux of the impossible narrative: being placed in a rules-oriented setting without knowledge of the rules can disorient the respondent and subtract from their own story.

As noted earlier, the history of US immigration restriction tells us that the law does not stop at presuming that non-citizens are threatening and/or deserving. It continues by subjecting a class of people to “rules that would be unacceptable if applied to citizens,” as the US Supreme Court decided in *Demore v. Kim* in 2003.^14^ These are the rules that the HRDP observations often cite as procedurally unfair. They turn away from the relational-based understanding of the law and emphasize how a respondent’s social inclusion is impossible because of their “illegal” status (Ngai 2004). However uncomplicated that may sound, the rules do not imply a straightforward and just legal process. An observation from a master calendar hearing demonstrates this issue:

[The respondent] had previously requested asylum, but had not filled out an application because [he] couldn’t find an attorney to help him. He had also been granted bond at a previous hearing. He had not paid it, because he was unable to contact his uncle in AZ for financial help. He stated that he had been removed from his home in AZ without his phone, and so had no contact info to reach the uncle. Judge asked Govt [DHS attorney] how this kind of situation could be helped, he had no answer. Judge explained that they could not release him without payment. He then asked to be deported. Judge asked how, if he was afraid of return. He is the sole support for his family and he is more afraid of them starving than of his fear for his life. They reluctantly ordered removal.

In this observation, a *pro se* respondent’s inability to pay bond leads to their removal order. While the IJ was mindful of the respondent’s fear of return, they still deferred to the court’s rules and procedure. Their deference penalized the respondent by acknowledging their personal story but denying a broader social truth, which is that there are conditions of acceptance and inclusion the respondent is financially incapable of fulfilling. Shut out from fully engaging in the court process, the respondent also had no means to seek help from their family. Because the law does not give the IJ enough discretion to amend those conditions, the respondent was unable to seek justice in their own trial. The social, logistical, and financial blockages preventing *pro se* respondents from developing and refining their own narrative strategy until the merits hearing is a feature, not a flaw, of immigration court today.

Another observation of an impossible narrative involves a *pro se* respondent from Burma. His family had legal permanent residency in the United States, though the respondent’s legal status was unclear to the court:

The [respondent] came to the United States as a refugee with his family. Parents had fled from Burma to Thailand, where he was born. He stated that his parents have applied for US citizenship but does not know their status or their birth dates : : : adjusted to LPR in August 2011. Government found a list of family members at their entry. These included his birth mother, who had died in Burma when he was five years old. So his “parents” were his father and his stepmother. Not known if he was adopted, so information on his entry was incorrect. Government couldn’t determine the status of his parents. The underlying question is—is respondent a US citizen? There is much confusion about his family.

While the final outcome of this case is unknown, it shows how liminal legal status can be and how in immigration court the impossible subject becomes statutorily ungovernable. The proposed solution rests not on the narrative that the respondent offers to the court—which is often formed by their liminal status and its impact on their social experience—but, instead, depends on whether or not they can prove themselves to legally remain in the country. This refers back to how the law articulates territory and sovereignty and how bureaucracies often fail to see beyond a rule-based orientation of the law. In the aforementioned example, this failure occurred partly due to the DHS’s own improper adherence to the rules, as the judge in this case was very frustrated that the prosecution’s evidence was incomplete and that they “couldn’t supply the needed information.”

Impossible narratives also emerge from the immigration court process itself. Court procedures can vacillate between abrupt adjudication and long, drawn-out exercises in rescheduled hearings, technological delays, and postponed decisions. Because hearings can move quickly, this can lead to little or no interaction between the respondent and the IJ. In one master calendar hearing where the defense attorney failed to show up, “[it] took so little time that the judge had very little contact with the detainee.” As such, it became difficult or irrelevant in that miniscule fraction of time for any party to work through the respondent’s circumstances and privilege a relational-based orientation of the law. A case can also slow down: sometimes, IJs grant continuances elongating the time allowed to find an attorney and gather pending documents, as our observations indicated. IJs would usually grant their request for a two-to-three-week extension, though this often came with a stern warning that they may not grant additional continuances in the future. In another master calendar hearing involving a Honduran respondent, an observation points out that the detainee had asked several times for an extension and had not been able to find an attorney. The judge commented she was aware of his physical limitations, without going into any detail, and has been asking that he be provided with an attorney who can represent him.

For those with representation, their attorneys would sometimes request an additional continuance from the judge in order to better prepare for trial. As one observation recalls, a lawyer who was hired earlier in the day immediately sought a rescheduled hearing for both bond and removal hearings so that they could “have time to learn the case.” In another case, a respondent had not yet met their new attorney, and while they were ready to defend their client during the bond proceeding, the DHS attorney was missing important hearing documents due to a recent government shutdown. During the court process, both sides of counsel are often left short-handed in understanding the nature of the case, and often do not see or meet the respondent prior to trial. Meanwhile, the respondent must navigate the anachronistic nature of their trial, while still remaining at the ready to tell a story in a moral, chronological order suitable for the court’s understanding.

While many of these hearings may be short, the length of detention is more interminable, which can preclude respondents from fully engaging in the court process. Observers mentioned the effect that the rules of mandatory detention can have on a case’s trajectory, leading to extended separation from families and severance from basic sources of social and economic support, including a potential loss of income. In some cases, detention can also lead to prolonged detachment from the communities and families who support and rely on them. In a few cases, detainees simply asked the judge to deport them on the spot. One Honduran respondent “repeatedly stated [their] desire to go home,” as one observer puts it, but the judge “repeatedly explained the need to follow court procedure while reassuring the detainee that he’d be deported as soon as the process had been completed.” Similarly, another bond hearing observation notes a Mexican respondent who emphatically repeated: “I want my deportation right now.” The judge had to repeat and clarify questions and rights. The detainee finally understood that the judge just wanted his acknowledgment of his rights before issuing the order for removal.

As this example shows, there can be breaks in communication between the IJ and respondent as well as a break between rule- and relational-based orientations toward the law. Even when the respondent wants to be deported, the law requires an adherence to process as an articulation of an individual’s rights, as limited as they may be. So while immigration court often appears inefficient in terms of time, scheduling, and pace, it also embraces bureaucratic state control and the rule of law to maintain a sense of order. Immigration court process relies on these rules in order to subject respondents to an “arbitrary, disproportionately harsh system” that privileges a non-citizen’s exit over their stay (Kanstroom 2007, 15).

This lack of engagement also becomes clear when observing everyday interactions between bureaucratic state actors (such as the IJ or ICE prosecutor), non-state actors (such as an interpreter or defense attorney), and the respondent. With so many legal actors in one room, different points of view may obstruct or privilege certain narratives in court (Conley and O’Barr 1990). For instance, an IJ’s demeanor can sometimes prevent them from seeing beyond the respondent’s personal story and consider how court processes may limit a respondent’s ability to engage during proceedings. One IJ was “rude and abrupt” to a Sudanese respondent who had just retained counsel the day before and asked for a continuance. During another master calendar hearing, a different IJ was “impatient with [the] respondent’s lack of understanding of rights and legal processes.” Another observation notes that, for a bond hearing, the same IJ was “frustrated that the detainee was not prepared with an attorney”—as the observation notes, the respondent had failed to tell his attorney to show up.

These frustrations can signal a broken process, and they can also create tension in the courtroom. Another observation involved a master calendar hearing for an unrepresented Cuban respondent who first wanted his case transferred closer to his family, who could not afford to travel to Minneapolis; he then “talked about wanting deportation.” This observation pointed out that the judge was “abrupt,” interrupting a *pro se* Cuban respondent who then became visibly upset as evidenced by his face becoming flushed, his voice cracking, and tears welling in his eyes. When the judge was talking to him, they were shuffling and stapling paperwork at the same time. These interactions show how impossible narratives can further entrench ideas about not only who belongs in the United States but also who is able to engage in the legal process to begin with. Moreover, they emphasize Conley and O’Barr’s (1990, 176) claim that “the legal thinking of lay people is neither random nor illogical” but that such thinking is “structured according to different precepts than the rule-oriented thought process that dominates legal reasoning.” For instance, in the absence of an attorney, an observer writes, a respondent in a master calendar hearing “was engaged, but arguing legal issues that didn’t apply to this type of hearing.” In another case, a respondent confused his master calendar hearing with his bond hearing when his attorney was absent. The respondent then asked for the hearing to be delayed so that he could gather more evidence. This example highlights the deleterious consequences of navigating legal status alone in court, which limits the respondent’s ability to understand court procedure and tell their story in the process.

The IJ and ICE prosecutor’s body language and tone can be abrupt, impatient, and frustrated following a respondent’s testimony that they may perceive as extraneous. In these scenarios, one sees how legal authority can immediately dismiss any narrative that exists outside of legal precepts; in short, legal authority gets to decide what narratives are irrelevant and outside the purview of the rules. Disregarding the respondent’s story as undeserving of due process assumes a “taken for grantedness” of the law that denies the humanity of a Hmong respondent, as one observation of a master calendar hearing makes clear: “No eye-contact from judge to detainee. Why? So dehumanizing. He is afraid to return to Laos because he doesn’t have documents to live there nor land to farm or survive. He came to the U.S. in 1992 as a refugee. Has 6 kids. Judge asked for no talking from myself and [another] observer after I rustled a paper and he asked if I had a packet.”

This observation highlights how the tension between rule- and relational-based orientations toward respondent’s stories and even highlights the observer’s own presence in the courtroom. While, in some cases, the judges were “kind,” “compassionate,” “respectful,” “professional,” and “fair,” this relational-based understanding of court process shows how deservingness narratives, like threat narratives, develop from interactions between the law’s facilitators and the non-citizen on trial and that relational aspect can often overlook aspects of a story that non-legal actors may consider to be very important.

Legal interpreter services also contribute to impossible narratives. Observations note how IJs are selective in their attempts to bridge between language barriers and work through interpreter issues. In many instances, judges try their best to assist respondents in this regard: one observer called the judge “respectful” and pointed out that they asked if the respondent wanted to request a Quiché interpreter for their bond hearing. In other situations, judges are less patient. Another observation points to a bond hearing where “a judge said it’s too cumbersome to have everything translated so they did summaries and had those translated. Seemed really unfair to the detainee.” In another hearing (also for bond determination), the respondent’s attorney called into the trial via telephone, and a “slight language barrier” kept the detainee from fully understanding the proceedings. “Her Chinese interpreter would be in the office soon,” the observer pointed out, “and they would call the detainee together to make sure he understood everything.”

Another example shows more generally how interpreter issues can lead to further delays and confusion:

In the September 18 initial removal hearing, five to ten minutes were spent on obtaining a phone Hmong interpreter. None were available from the two resources tried by [the judge]. She postponed his hearing to “tomorrow.” I am not sure what happened with the scheduling of the hearing, but when I came for another observation shift on September 25, this detainee was having his hearing (with a Hmong phone interpreter); I don’t know if this was a result of several more “tomorrow” postponements because of an unavailable interpreter, or if it was a reschedule based on something else.

Language and interpreter challenges remain important due process issues, and they also underscore how difficult it is for the respondent and other legal actors to translate narrative and promote understanding of the law. Some observations point out that some detainees did not have the proper language resources to help them file their paperwork, with one observer asking: “How can detainees complete [an] application when they are in jail and speak little to no English?” Without dependable interpretation services, unrepresented non-citizens, in particular, are immediately constrained in their ability to participate in a meaningful and engaging way. These bureaucratic failings—from time lapses to literal misinterpretations of respondents’ stories and understandings of the law—create a legal space that renders respondents’ engagement with the state both impractical and impossible. The court process leaves them excludable, unable to fully participate, and without means to stay in the United States.

## DISCUSSION AND CONCLUSION

While US immigration court appears on the brink of collapse, we contend that it continues to function today due to the power and social control of crimmigrating narratives. Our analysis of courtroom observations points to three narratives (threat, deservingness, impossibility) rooted in statutory provisions that in turn reflect social and political discourse, both current and historical. And while these framings do not always align with lay understandings of the law, third-party observations highlight how they move the legal process forward and disassociate non-citizens from their social realities.

Building on Ewick and Silbey’s (1995) sociology of narrative, we argue that these three dominant narratives privilege particular truths that the respondent and other legal actors can tell in immigration court. Meanwhile, these narratives discount broader, more structural truths about the US immigration system today, which are that it lacks due process and targets certain immigrant groups as a means of social control. These narratives encourage a “taken for grantedness” that ignores any connection the respondent’s story may have to more generalized social experiences of being a non-citizen in the United States, belonging to a particular origin group, and being potentially without legal status. Taken together, these conditions demonstrate how the law in action often regenerates existing relations of power and criminalizes the respondent even further. The law’s tight grip on discretion means that respondents’ stories must appear personal, unique, and not only convincing enough to the IJ in order to grant relief but also within the bounds of specific criteria written in codified law. In doing so, crimmigrating narratives facilitate a connection between personal story and the relational structure that make their story visible in court, but they also purposefully ignore how the conditions of their existence are formed in part by larger, more global power structures, such as the law, which remain rigid in an increasingly interconnected and migratory world.

A key contribution of this article finds that respondents’ own stories often fail to fit into those narratives and that the institutional power of the law often rejects their stories by default. In this sense, courtroom observations can help to reveal how legal process can decontextualize, abridge, and even disregard personal narratives as untruthful and, by doing so, dispossess experiences of their meaning (Barthes 1975; Polkinghorne 1988; Bruner 2004). Our results demonstrate how court process effaces the connection between the respondent’s personal story and a broader, more complex relationship to their social experience. With that in mind, another theoretical contribution of this article is to show how competing narratives in the immigration court context engrain the relational binary of the “haves” and “have nots,” which point to the powers and privileges of citizenship as an institution.

Our findings also reveal the strategy behind crimmigrating narratives and who has access to producing countervailing narratives in court. During cross-examination in a removal trial, ICE prosecutors are strategic in locating the threat, deservingness, and impossibility of the respondent’s ability to remain in the United States. If the respondent has an attorney—in other words, a “strategist” who helps them choose what story to tell—they can downplay such arguments, but there are many instances in our sample where *pro se* respondents must initiate a narrative strategy on their own. This can be difficult for the respondent, as past work demonstrates that having counsel in immigration court increases the likelihood of a successful outcome (Eagly and Shafer 2015; Ryo 2018, 2019a, 2019b). Speaking on why retaining counsel may lead to more successful case outcomes, Ryo (2019a, 246) writes that “one possibility is that lawyers advance personal or individuating information about their clients that makes it difficult for immigration judges to engage in simple heuristics or categorical thinking about detainees as dangerous criminals.” Our results add to this idea by showing how easy it becomes for adjudicators to accept crimmigrating narratives at face value at the start of a trial. This often occurs without another narrative strategy to displace it, especially when *pro se* respondents have little to no guidance on how to prepare their case before a judge.

We note in our results how some elements of a respondent’s story can overlap with logico-deductive application of the law—for instance, if they argue that their deportation would cause undue harm to their US citizen child left behind, then the court might interpret it to be a violation of that child’s constitutional protections. But if their child is not a US citizen or a green card holder, then that nexus connecting social experience and personal story disappears, or, at the very least, the legal status of their relation makes the respondent lawfully ineligible for relief. Our results also show that, despite their core distinctions, the three crimmigrating narratives that we have outlined are bound and reinforcing toward one another; they are not separate entities vying for attention. Instead, we have found that, by taking these themes together, one can identify and analyze how civil procedure turns detention and removal into administrative, punitive, criminalizing processes. As such, deservingness narratives’ moral logic for inclusion complements threat and impossible narratives’ logic for exclusion. This observation resonates with Asad Asad’s (2019) findings that IJs in the Dallas immigration court avoid the removal process for those they consider deserving of relief, while, in other cases, IJs rely on a “scripted approach” of well-rehearsed narratives for those they deem as impossible to circumvent deportation. Our results add to this dialogue on courtroom narratives by showing how the law and legal processes limit discretion based on rules that repeat and affirm respondents’ exclusion.

Another contribution is our emphasis that all three crimmigrating narratives place the burden of proof on the respondent detainee. Non-citizens’ stories are often personal but contained in a legal space. Stories of respondents’ dreams, commitments, and desires are powerful in the legal sense only if they are perceived as non-threatening, deserving, and/or “possible subjects.” To borrow from Ngai (2004, 5) once more, respondents in court must fight to avoid perceptions of being “a person who cannot be and a problem that cannot be solved.” This idea, on the one hand, not only tends to disengage the respondent from the process but also formulates a retelling of their story in an order perceived as cohesive, competent, and chronological. But, on the other hand, even with attorney representation to aid them in strategic storytelling, we find that the immigration court process often re-criminalizes respondents in civil proceedings. Crimmigrating narratives reference their criminal history to (1) prove their threat to society; (2) privilege moral justifications that determine their deservingness to belong in the United States; and/or (3) rely on the law as written to make those non-citizens “illegal.” These forms of social control, which serve various gatekeeping functions, are on full display in the HRDP volunteers’ observations of the Fort Snelling immigration court. Furthermore, our results highlight how important it is to understand that non-citizens today are formed and reformed via the structural conditions of narratives and that immigration court uses them to force individuals into certain patterned social positions (Emirbayer 1997; Bourdieu and Wacquant 1992).

The socio-legal scholar Susan Silbey (1991, 430) said that “ultimately law is a matter of force, but it is also a matter of words, words that enable the state-sanctioned use of force.” In the context of immigration court and the crimmigrating narratives, those words do not always comport with lay understandings of law and justice. Third-party observers’ perspectives show the lack of resolution between a respondent’s personal story and the structural power relations that entail the US immigration system. We note that it is difficult for respondents to tell their story in a court process that ignores broader social experiences of immigration enforcement. Additionally, the court process’s own dysfunction and delay can inhibit the respondent’s ability to formulate a narrative over multiple hearings, leading to an asynchronous retelling of their lives without an appreciation of their social context.

Nearly forty years ago, immigration legal scholar Peter Schuck (1984, 1) wrote that “immigration law remains the realm in which government authority is at the zenith and individual entitlement is at the nadir.” To that end, not much has changed in the US immigration system. The historical legacy of immigration restriction—from slavery to the Chinese Exclusion Act to targeting so-called “illegal aliens” today—demonstrates that, while immigration courts may be on the brink of collapse, it has always been part of a larger system intent “to punish, stigmatize, and marginalize” (García Hernández 2019, 13).^15^ In that vein, this article speaks to how the current structures of immigration law use crimmigrating narratives in court to justify non-citizens’ punishment in a non-punitive setting. More and more, it is becoming the job of researchers and public observers to work within and call attention to this realm and to support advocates and legal practitioners who have already begun to open pathways of understanding into the immigration court system and its procedures. Our hope is that future research explores third-party observations further, as they can elicit public understandings of detained and non-detained immigration court where crimmigration continues to expand. Such work can continue to investigate what stories are told in court and what stories get lost in the law.

## Supplementary Material

Court Observation Form

## Figures and Tables

**TABLE 1.** Case and respondent characteristics stratified by judge (observation level)

	Judge Option 1 (%)	Judge Option 2 (%)	Judge Option 3 (%)
	(n = 124)	(n = 123)	(n = 54)
Representation			
Pro se (no attorney)	33.06	31.71	35.19
Attorney appeared in person	61.29	60.16	57.41
Attorney appeared by phone	5.65	8.13	7.41
Hearing type			
Master calendar	38.71	27.64	18.52
Bond	36.29	35.77	35.19
Combined master/bond	16.13	30.08	37.04
Unknown	8.87	6.50	9.26
Interpreter used	57.26	60.16	61.11

## Footnotes

1. Multiple reports stated concerns about Executive Office for Immigration Review’s (EOIR) bandwidth regarding technology, including their reliance on paper-based filing, faulty equipment, lackluster Internet connectivity, and inadequate telephonic interpreter services. See American Bar Association 2019.

2. Anti-Drug and Abuse Act, October 27, 1986, 100 Stat. 3207; Illegal Immigration Reform and Immigrant Responsibility Act, April 1, 1997, 110 Stat. 3009–546.

3. One of the Biden administration’s actions in 2021 involved replacing the use of the word “alien” in EOIR decisions with more current terminology, such as “non-citizen,” “respondent,” “petitioner,” and so on. See Department of Justice 2021b.

4. In the criminal justice context, Patricia Ewick and Susan Silbey (1995, 215–16) refer to the US Supreme Court decision in *McCleskey v. Kemp* (481 U.S. 279 (1987)), in which the court upheld the death penalty sentencing of Warren McCleskey (a Black man) for armed robbery and murder despite statistical evidence submitted to the court of racist bias in death penalty sentencing more generally; in Ewick and Silbey’s words, such evidence “was simply not sufficient to establish that any racial discrimination occurred *in this particular case*” (emphasis in original). In their decision, “the court insists on a narrative that effaces the relationship between the particular and the general, between this case and other capital trials in Georgia” (Ewick and Silbey 1995, 216).

5. In her analysis of US immigration restrictions from 1924 to 1965, Mae Ngai (2004, 4) writes that certain non-citizens’ inclusion in the nation “was simultaneously a social reality and a legal impossibility.” While many non-European immigrants were deported during that time, administrative discretion granted many “illegal” European immigrants the right to stay (De Genova 2002).

6. Some of these projects include: the Atlantans Building Leadership for Empowerment in Atlanta; the Chicago New Sanctuary Coalition and the Immigration Court Watch Program in Chicago; the Immigrant Detainee Justice Project in Seattle; and the Immigration Court Observation Program in New York, San Francisco, and Seattle.

7. The HRDP website is accessible at https://perma.cc/K9ZW-3A8H.

8. The HRDP developed this form as the Fort Snelling court was transitioning out of a recent immigration judge (IJ) hiring process. As the court infrastructure evolved substantively and mechanically, observers indicated three options on the form: Options 1 and 2 were courtrooms with one IJ each, while Option 3 involved another courtroom where one IJ was in the process of retiring and another was in the onboarding phase.

9. Also per EOIR guidelines, IJs have the authority to close an immigration hearing at any time and ask any court visitor to identify themselves and their purposes for attending trial.

10. While the “immigrant gateways” literature has primarily focused on the sheer numbers of foreign-born individuals living in a given state, we also pay attention to the growth rate of the foreign born. States comprising the Upper Midwest region (Iowa, Minnesota, Nebraska, North Dakota, and South Dakota) have all experienced a disproportionately large change in the immigrant population between 2010 and 2016 (Batalova and Alperin 2018).

11. This example highlights the recent US Supreme Court decision of *Padilla v. Kentucky* (559 U.S. 356 (2010)), where the petitioner claimed that his criminal attorney gave him bad advice upon entering a plea bargain for a drug-related charge. The court decided henceforth that criminal defense attorneys are required to advise non-citizen clients about the deportation risks that come with a guilty plea in criminal proceedings.

12. Rules of evidence also generally differ from the criminal court system, where both the prosecution and defense obtain copies of the evidence that the other side has gathered. In immigration court, prosecutors are not required to share their evidence; instead, they selectively choose what enters the record after the respondent makes their statements in a hearing (Heeren 2013).

13. Following a Department of Justice memo in October 2019, multiple driving-under-the-influence convictions are “strong evidence that an alien lacked good moral character during that time and is thus not eligible for cancellation of removal.” See *Matter of Castillo-Perez*, 27 I&N Dec. 664 (A.G. 2019).

14. *Demore v. Kim*, 538 U.S. 510 (2003).

15. Chinese Exclusion Act, May 6, 1882, 22 Stat. 58, c. 126.
