# Supplementary material for: Crimmigrating Narratives: Examining Third-Party Observations of US Detained Immigration Court
Source: Law Soc Inq. Author manuscript; Available in PMC 2024 May 1. (PMC10732548; doi:10.1017/lsi.2022.16)
Supplement: Court Observation Form [file NIHMS1905591-supplement-Court_Observation_Form.pdf]

Date:______________________ Observer:____________________________

Human Rights Defender Project: Court Observation Form

General

1. **Last 3 digits of A#:**____________
2. **Immigration Judge** ☐ [redacted] ☐ [redacted] ☐ [redacted]
3. **Government Attorney Name**: ______________________
4. **Was the detainee represented?** ☐Yes, in person ☐Yes, by phone ☐No
    a. **If yes, what type of attorney?** ☐Private ☐Non-private (NGO/volunteer) ☐Unknown
5. **Detainee's country of origin**: ____________________________
6. **Gender** ☐Male ☐Female ☐Transgender and/or non-binary
7. **Type of hearing** (if more than one, check both):
    ☐Bond ☐Continued Bond ☐Initial Removal Hearing ☐Continued Removal Hearing ☐ Unknown
8. **Detainee's entry date/length of time in the US** (if known): ____________________
9. **Does the detainee have family in the US?** ☐ Yes:____ ☐ No

Language

10. **Detainee's preferred language:** ___________________________
11. **Estimate English fluency of detainee**: ☐ Fluent ☐ Proficient ☐ Very little/none
12. **Was an interpreter used?** ☐ Not needed ☐ Physical interpreter ☐ Phone interpreter ☐ No interpreter available
13. **Were there any technical issues with interpretation?** (hearing/equipment issues, phone was dropped etc.) If yes, please describe: ____

____

Mental Health

14. **Was there any mention of the Respondent having any mental health issues?** If yes, describe what was mentioned, whether the Judge or the court required ICE to do anything, or if the Judge indicated they would try to have an attorney take the case: ____

____

15. **Do you think Respondent may have a mental health issue or cognitive impairment? If so, why?**

____

____

Criminal History

16. **Was a criminal history mentioned by either the court or lawyers**? If so, list what you can. Please indicate if charge is pending, conviction, dropped, or acquitted: ____

____

____

Bond Hearings – skip if no bond hearing was requested or conducted

17. **Was bond agreed between parties prior to hearing?** If yes, skip remaining Bond questions ☐ Yes, $______ ☐No

Bond - continued

18. **Was bond requested**? ☐ Yes ☐ No, continuance ☐ No, withdrew bond request
    a. **If bond was requested, did the Judge grant it**? ☐ Yes, $__________ ☐ No
        i. **Did DHS reserve appeal**? ☐ Yes ☐ No
    b. **If bond was denied, why?** ☐ Ineligible/mandatory detention ☐ "danger to society" ☐ "flight risk" ☐ Other:____________________
19. **What factors, if any, were mentioned in granting bond?** ____________________

Removal Hearings - skip if no removal hearings were conducted

20. **Were pleadings taken?** ☐ Yes ☐ No
21. **Did the detainee ask to be ordered removed?** ☐ Yes ☐ No, asked for continuance ☐ No, asked for relief
22. **If the detainee requested relief, what type of relief?** (includes application forms)
    ☐ 42A (LPR) Cancellation ☐ I-589 Asylum ☐ Adjustment of Status
    ☐ 42B (non-LPR) Cancellation ☐ I-589 Convention Against Torture (CAT) ☐ U-visa or T-visa
    ☐ Voluntary Departure ☐ I-589 Withholding of Removal ☐ Other/unsure:__________
23. **Did the Judge order removal/deportation?** ☐ Yes ☐ No **Did the Judge grant Voluntary Departure?** ☐ Yes ☐ No
24. **Did the Judge order a continuance?** ☐ Yes, next hearing date: __________ ☐ No

Overall Impressions

25. **Did the Judge take time to explain what was happening** (especially if the detainee was not represented)? ☐ Yes ☐ No
26. **Did the Judge take time to answer questions**?
    ☐ Yes ☐ No (detainee was not represented) ☐ No (detainee had an attorney)
27. **Do you believe the Judge treated everyone in court with respect?** ☐ Yes ☐ No
28. **Comments about the Judge (demeanor, attitude, respectfulness, patience, etc.):** ____________________
29. **Was the government attorney respectful during the proceedings?** ☐ Yes ☐ No
30. **Did the detainee's attorney interact with the client**? ☐ Yes ☐ No Details: __________
31. **Did the detainee's attorney provide good representation to their client?** ☐ Yes ☐ No
32. **Did the detainee appear to be engaged in the process**? If no, why not? ☐ Yes ☐ No
33. **Did the detainee have supporters in Court for them**?
    ☐ Family ☐ Other supporters ☐ Couldn't tell ☐ None
34. **How long did the hearing last?** ☐ less than 5 minutes ☐ 5-10 minutes ☐ 10+ minutes

Additional notes: ____________________

Case Referral (if so, please see appropriate online form): ☐ For representation ☐ Official complaint
